# mHealth Use, Preferences, Barriers, and eHealth Literacy Among Patients With Inflammatory Bowel Disease: Survey Study

**DOI:** 10.2196/64471

**Published:** 2025-11-13

**Authors:** Christopher Kretzschmar, Johannes Knitza, Robert Pietschner, Raja Atreya, Markus Friedrich Neurath, Till Orlemann

**Affiliations:** 1 Department of Internal Medicine 1 University Hospital Erlangen Friedrich-Alexander University Erlangen-Nürnberg Erlangen Germany; 2 Deutsches Zentrum Immuntherapie Friedrich-Alexander University Erlangen-Nürnberg and University Hospital Erlangen Erlangen Germany; 3 Department of Internal Medicine 3 University Hospital Erlangen Friedrich-Alexander University Erlangen-Nürnberg Erlangen Germany; 4 AGEIS Université Grenoble Alpes Grenoble France; 5 Institute for Digital Medicine University Hospital of Giessen and Marburg Philipps University Marburg Marburg Germany

**Keywords:** eHealth Literacy Scale, mHealth, mobile apps, inflammatory bowel disease, Crohn disease, ulcerative colitis

## Abstract

**Background:**

Mobile health (mHealth), defined as health care facilitated by mobile devices, offers a promising strategy for enhancing disease management and treatment for patients with chronic conditions. However, there is limited information about how patients with inflammatory bowel disease (IBD) use mHealth and their digital preferences.

**Objective:**

The aim of the study was to investigate the use of mHealth as well as the preferences, obstacles, and eHealth literacy reported by patients with IBD in Germany.

**Methods:**

In April and May 2023, we sequentially enrolled patients diagnosed with IBD, including Crohn disease and ulcerative colitis, to participate in a paper-based survey. The survey included questions on sociodemographic details, health characteristics, mHealth use, internet use, eHealth literacy (measured with the eHealth Literacy Scale), and preferences regarding communication and information.

**Results:**

Of the 200 surveyed participants, almost all (197/200, 98.5%) reported regular smartphone use, and more than two-thirds (139/200, 69.5%) indicated regular engagement with social media. Most of the respondents (168/200, 84%) expressed the belief that incorporating medical apps into their routine could positively impact their health. However, only 25 (12.5%) of the 200 patients acknowledged using medical apps, of which just 2 apps were IBD specific, used by only a few (n=3, 12%). Furthermore, awareness of useful websites or mobile apps tailored for IBD was limited (45/200, 22.5%). Nearly all participants (196/200, 98%) expressed willingness to share app data for research purposes, and most (171/200, 85.5%) consented to transmit app data to their treating physicians. A large majority (175/200, 87.5%) indicated readiness to regularly input data into an app, with a preferred duration of up to 5 minutes (109/200, 54.5%) and weekly input frequency (76/200, 38%). For an IBD-specific app, the most frequently requested functions were electronic prescriptions (110/200, 55%) and a newsletter about new scientific work and clinical studies (94/200, 47%). Usability and security were identified as key app attributes. The internet was the predominant source of health-related information (180/200, 90%). The average eHealth literacy score, measured with the eHealth Literacy Scale, was high (mean 28.9, SD 5.4; range 8-40), with a positive correlation observed between higher eHealth literacy and factors such as younger age and more frequent internet use for health information.

**Conclusions:**

Patients with IBD are well prepared and motivated to use mHealth technologies to better understand their chronic condition and optimize treatment. However, their enthusiasm is tempered by the currently low adoption of mHealth. To fully harness the potential of mHealth in IBD treatment, effective and tailored mHealth solutions, guidance for their implementation, and patient education are needed.

## Introduction

### Background

Inflammatory bowel diseases (IBDs), including Crohn disease and ulcerative colitis, are complex, chronic conditions with multifaceted pathogenesis [1,2]. Patients typically require lifelong treatment from a multidisciplinary specialist team, including gastroenterologists, surgeons, rheumatologists, dermatologists, and radiologists [3]. This care includes, apart from regular medical appointments, immunosuppressive pharmacotherapy to maintain remission and prevent relapses [4], which can pose challenges for patients’ treatment adherence [5]. The complexity of IBD not only incurs high health care costs [2] but also makes it difficult for patients to understand their illness and manage daily demands, such as monitoring symptoms, implementing appropriate nutrition, and planning activities [6]. Consequently, IBD represents both a substantial economic burden and a patient-level challenge, underscoring the need for modern disease management strategies [3,7].

In today’s world, the use of digital media such as computers, smartphones, tablets, the internet, and mobile apps is ubiquitous and plays an important role in the daily lives of many people. One area increasingly affected by these technologies is health. The internet serves as a gigantic source of information, making health information widely accessible; fitness trackers enable individuals to record and document personal health parameters; and digital services such as email allow patients to communicate with health care providers. Consequently, digital technologies are increasingly incorporated into routine health care [8-10]. The concept of mobile health (mHealth), defined by the World Health Organization as “medical and public health practice supported by mobile devices, such as mobile phones, patient monitoring devices, personal digital assistants (PDAs), and other wireless devices” [11], is a promising approach for managing various chronic diseases, including rheumatoid arthritis [12], diabetes mellitus [13], hypertension [14], and chronic heart failure [15]; for example, electronic medication reminders can improve treatment adherence [16,17], and supportive digital therapy can reduce pain [17] and improve key comorbidities (eg, depression) [18]. In the context of IBD, mHealth has the potential to take disease management to a new level, including improving patients’ quality of life and reducing health costs [19]. However, access to mHealth solutions remains extremely limited for patients with chronic IBD [20].

### Objectives

The perspectives of patients with IBD on mHealth solutions remain largely unexplored. To effectively incorporate various mHealth solutions into clinical practice, it is essential to understand patients’ limitations and needs. This study aimed to examine the use of mHealth solutions as well as the preferences, obstacles, and eHealth literacy reported by patients with chronic IBD in Germany.

## Methods

### Paper-Based Survey

In April and May 2023, consecutive patients treated at the outpatient clinic for chronic IBD at the University Hospital Erlangen were invited to complete a paper-based survey. This format was intentionally chosen to minimize selection bias by enabling participation regardless of digital literacy or device access.

To create the survey (Multimedia Appendix 1), a broad literature review was performed. Previous mHealth patient surveys in rheumatology served as a starting point [21]. The survey comprised four main sections: (1) sociodemographic and health characteristics, (2) preferences and use of mHealth, (3) eHealth literacy, and (4) communication and information preferences. Foreign terms and explanations of technical terms were provided in footnotes. Inclusion criteria were as follows: patients had to (1) be aged ≥18 years, (2) be proficient in the German language, (3) be physically and mentally capable of completing a structured questionnaire, and (4) have a confirmed diagnosis of either Crohn disease or ulcerative colitis.

The sociodemographic and health characteristics included age, sex, place of residence, educational qualification, diagnosis, duration of illness, patients’ global self-assessment of disease activity, and current use of electronic devices (smartphones, tablets, and activity trackers) and social media.

The section on preferences and use of mHealth contained questions about the use of medical apps in general, knowledge and use of IBD-specific apps, and the preferred duration and frequency of actively using an IBD-specific app. Patients were asked to evaluate their preferences for app features on a 5-point Likert scale and the importance of app characteristics on a 10-point Likert scale. In addition, patients’ willingness to share recorded app data and overall perceptions of the benefits of medical apps were investigated.

Furthermore, information was gathered on internet use and the perceived utility of the internet regarding health decisions, as well as on web-based services to enhance patient health and on telemedicine, with a focus on patients’ digital interaction preferences.

Measurement of the patients’ eHealth literacy was carried out using the validated German version [22] of the eHealth Literacy Scale (eHEALS) [23]. This tool has been translated and validated in multiple languages [23-28] and enables systematic capture of differences in eHealth literacy levels. It consists of 8 statements regarding eHealth literacy that are rated on a 5-point Likert scale ranging from 1 (“strongly disagree”) to 5 (“strongly agree”), yielding a total score ranging from 8 to 40.

Using ranking lists (unique rankings were required), patients indicated their preferences regarding medication reminders, the format of medical information, the design of digitally provided information, the type of patient diary, and the mode of physician communication.

### Statistical Analysis

The examined characteristics were summarized using counts, percentages, means, and SDs. To investigate relationships between continuous variables, Pearson correlation was used. The relationship between the eHEALS score and the frequency of internet use was investigated using the Jonckheere-Terpstra test, with eHEALS score as the continuous dependent variable and internet use frequency as the ordinal independent variable. Relationships between the eHEALS score and binary preferences were examined using logistic regression. The applied statistical tests were carefully selected according to the measurement scales and distributional characteristics of the collected data. All models included adjustments for age and sex. Two-tailed *P* values <.05 were considered statistically significant. Microsoft Excel and SPSS (version 28.0; IBM Corp) were used for data processing and analysis.

### Ethical Considerations

This study received approval from the ethics committee of Friedrich-Alexander University Erlangen-Nürnberg (23-63-B) and was conducted in accordance with good clinical practice. Before inclusion in the study, all participating patients provided informed consent for participation, pseudonymized data storage, and processing of the collected data. All participant’s data were collected, stored, and analyzed in accordance with applicable privacy regulations. Personal information was pseudonymized, and confidentiality was strictly maintained throughout the study. Participating patients did not receive any form of financial or other compensation for their involvement in the study.

## Results

### Patient Characteristics

A total of 220 patients were recruited for the study. For the final data analysis, only complete surveys from patients who met all inclusion criteria (200/220, 91%) were considered. The number of patients who declined participation was not recorded. The sociodemographic data of the study sample are presented in Table 1. The mean age was 39.1 (SD 14.0) years, with 49% (98/200) of the patients aged between 18 and 35 years. Sex distribution was nearly balanced. Regarding the use of electronic devices and social media, almost all patients regularly used a smartphone (197/200, 98.5%), more than half (108/200, 54%) regularly used a tablet, nearly one-third (64/200, 32%) used an activity tracker, and more than two-thirds (139/200, 69.5%) regularly used social media.

**Table 1:** Demographic and health characteristics (n=200).

Characteristics			Participants
Age (years), mean (SD)			39.1 (14.0)
**Age (years), n (%)**			
	18-35	98 (49)	
	36-45	39 (19.5)	
	46-55	28 (14)	
	56-65	26 (13)	
	>65	9 (4.5)	
**Sex, n (%)**			
	Female	96 (48)	
	Male	104 (52)	
**Diagnosis, n (%)**			
	Crohn disease	127 (63.5)	
	Ulcerative colitis	73 (36.5)	
Patient global self-assessment of disease activity (on a scale ranging from 0 to 10), mean (SD)			2.3 (2.3)
Disease duration (years), mean (SD)			13.1 (9.9)
**Disease duration (years), n (%)**			
	≤1	8 (4)	
	2-5	46 (23)	
	>5	146 (73)	
**Place of residence, n (%)**			
	Village	95 (47.5)	
	Small city	56 (28)	
	Midsized city	18 (9)	
	Big city	31 (15.5)	
**Regular use of devices, social media, and apps, n (%)**			
	Smartphone	197 (98.5)	
	Tablet	108 (54)	
	Activity tracker	64 (32)	
	Social media	139 (69.5)	
	Medical apps	25 (12.5)	

### mHealth: Use, Knowledge, and Acceptance of Medical Apps and Willingness to Provide mHealth Data

A set of questions assessed patients’ preferences and attitudes toward potential mHealth apps and the sharing of health data. An overview of the collected data is shown in Multimedia Appendix 2. Regarding app use, only a small proportion of patients (25/200, 12.5%) reported currently using medical apps. Among these, only 2 apps were IBD specific: CED-Forum (2/25, 8%) and an unnamed IBD app (1/25, 4%). Patients’ knowledge of useful IBD-specific digital services (websites and apps) was generally low (45/200, 22.5%). Notwithstanding these findings, more than three-fourths of the patients (168/200, 84%) believed that using medical apps could be beneficial for their health. The potential association between eHEALS scores and the likelihood of expressing the belief that apps are beneficial was investigated using logistic regression adjusted for age and sex. It turned out that our model, using the eHEALS score as predictor, fit poorly, as indicated by the very low explained variance (Nagelkerke *R*^2^=0.09), suggesting that eHEALS scores alone account for little of the variation in patients’ perceptions of app benefits.

Almost all patients (196/200, 98%) indicated willingness to transmit app data for scientific research, on condition that data security was guaranteed. Similarly, most of the patients (171/200, 85.5%) agreed to share app data with their physician. Among those who did not agree (29/200, 14.5%), the main reason was the perception that personal contact with their physician was sufficient (17/29, 59%). Other reasons included concerns about not knowing where their data would be stored (12/29, 41%), unclear use of the data (11/29, 38%), and insecure data transmission (9/29, 31%). Whereas the majority of patients (188/200, 94%) wished to be notified if an app detected irregularities regarding their health, interest in comparing medication adherence with other patients was low (57/200, 28.5%).

**Figure 1:**
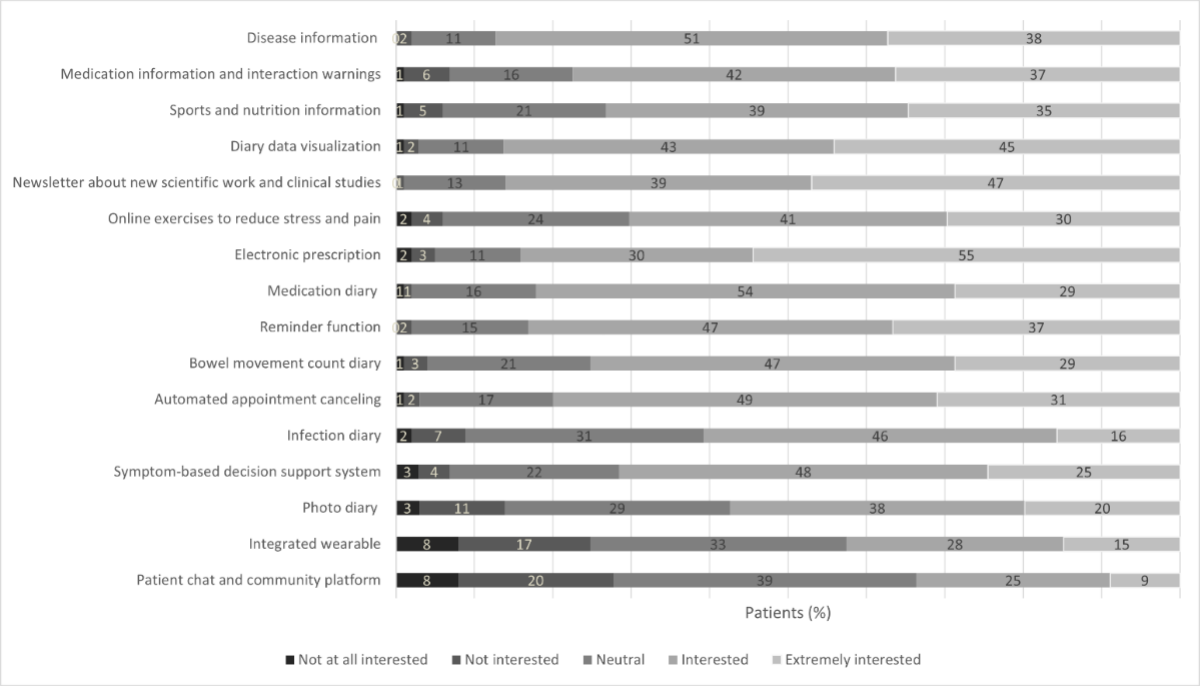
Patients’ app function preferences (responses to “What app functions would you like?”).

A large proportion of the participants (175/200, 87.5%) were willing to actively input data into an IBD-specific app. Among all patients, weekly data entry was preferred by 38% (76/200), with a duration of 1 to 5 minutes (109/200, 54.5%). When asked to evaluate key attributes of an app on a 10-point Likert scale, patients rated usability (mean 9.0, SD 1.4) and security (mean 8.4, SD 2.6) as the most important (Multimedia Appendix 2). Regarding preferred app features, patients were most interested in electronic prescriptions and a newsletter about new scientific work and clinical studies. By contrast, patients were least interested in direct peer exchanges, such as chats, or integrated wearables (Figure 1).

### Internet Use, Perceived Benefit, and Digital Interaction

**Figure 2:**
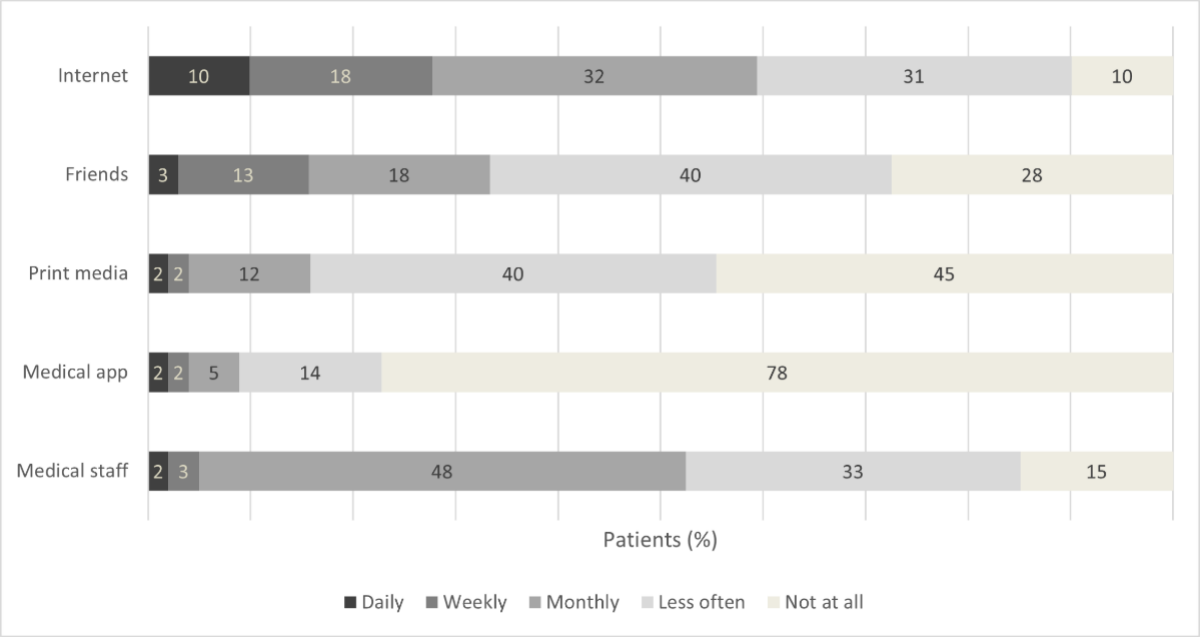
Use of different health information sources (responses to “How often did you use one of the following options to receive health information in the last three months?”).

Another important aspect of our study was to obtain knowledge about patients’ internet use, especially regarding health-related information. Almost all patients (193/200, 96.5%) stated that they had used the internet to seek health information, whereas only a small proportion (7/200, 3.5%) had not (Multimedia Appendix 3). The reasons for not seeking health information on the internet included perceiving their physician’s information as sufficient (4/7, 57%), considering internet searches unhelpful (3/7, 43%), and other reasons (1/7, 14%). The types of health information patients sought on the internet are illustrated in Table 2. Most patients searched for information about treatment options (165/193, 85.5%) and symptoms (164/193, 85%), whereas information on support groups was least frequently sought (85/193, 44%). Regarding the frequency of internet use for obtaining health information, the most common response was “less often than monthly” (79/200, 39.5%). Comparing the use of different health information sources during the past 3 months (Figure 2), the internet was the most frequently used source (180/200, 90%), with 10% (20/200) using it daily, 17.5% (35/200) weekly, 31.5% (63/200) monthly, and 31% (62/200) less often. More than half of the patients (116/200, 58%) had previously used online support groups to read information (113/200, 56.5%), chat with other patients (17/200, 8.5%), or post information (11/200, 5.5%). Participation in an online health program was infrequent (9/200, 4.5%), and knowledge about the medication website of the German competence network for IBDs was not widespread (29/200, 14.5%).

**Table 2:** Health information previously sought on the internet (n=193; responses to “What health information did you look for on the internet?”).

Health information	Patients, n (%)
Treatment options	165 (85.5)
Symptoms	164 (85)
Medication	158 (82)
Diagnosis	144 (75)
Physicians	142 (74)
Disease-specific websites	93 (48)
Support groups	85 (44)
Other	4 (2)

Regarding the perceived benefit of using the internet to make health-related decisions, results were ambiguous with approximately the same number of patients being unsure (81/200, 40.5%) and recognizing the internet as useful (79/200, 39.5%; Multimedia Appendix 3).

Another aspect investigated was patients’ digital interaction with their treating physician. Slightly more than half of the patients (105/200, 52.5%) reported having previously communicated with a physician via email. In addition, 71% (142/200) of the patients indicated that they would appreciate the option to communicate with their gastroenterologist via video call. The majority of the patients (159/200, 79.5%) favored electronically filling out medical questionnaires before their appointments, and more than two-thirds (137/200, 68.5%) preferred receiving an electronic medical report rather than a paper report.

### eHealth Literacy

**Figure 3:**
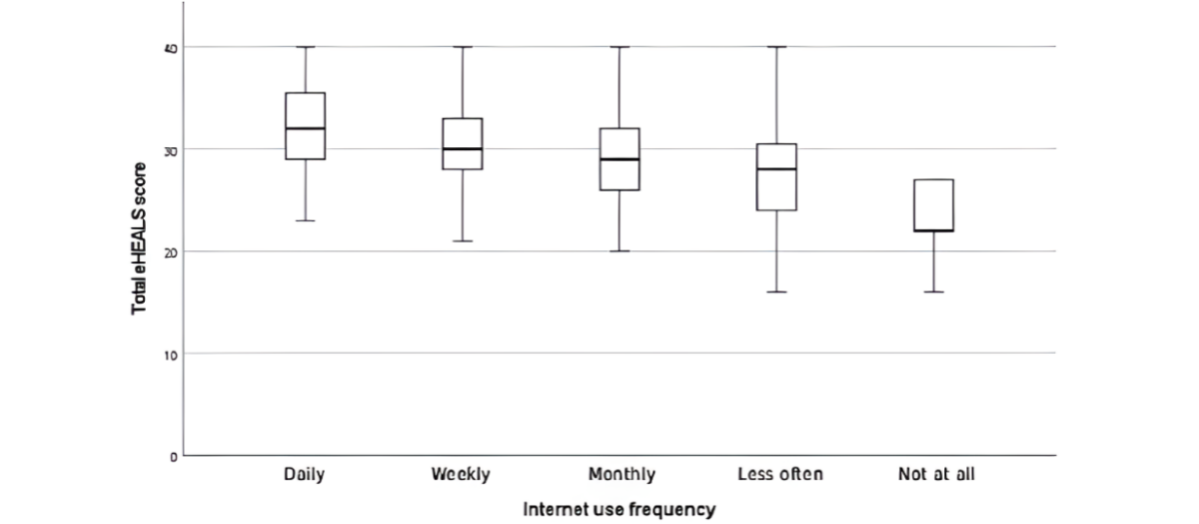
Association of eHealth Literacy Scale (eHEALS) score and frequency of internet use.

eHealth literacy, as defined by Norman and Skinner [23] is “the ability to seek, find, understand, and appraise health information from electronic sources and apply the knowledge gained to addressing or solving a health problem.” Therefore, eHealth literacy is an important dimension to record patients’ handling of digitally provided health information. The mean eHealth literacy score of our patients, measured using the eHEALS tool, was 28.9 (SD 5.4; range 8-40). Male patients had a mean score of 29.0, and female patients had a mean score of 28.3, resulting in a mean difference of 0.7; this difference was not statistically significant (*P*=.35), as verified by an unpaired, 2-tailed *t* test (95% CI −0.74 to 2.07). By contrast, age was found to correlate negatively (*r*=−0.2, 95% CI −0.35 to −0.04) with the eHEALS score. An overview of the distribution of responses to the 8 eHEALS items is provided in Multimedia Appendix 4. The majority of patients agreed or strongly agreed that they know how to use the internet to answer health-related questions (152/200, 76%) and have the skills to evaluate the health resources found on the internet (160/200, 80%); however, concerns were raised regarding the knowledge of available health resources on the internet (41/200, 20.5), and approximately one-third of the patients (69/200, 34.5%) expressed discomfort about using information from the internet to make health-related decisions. Lower eHEALS scores were found to be associated with a declining frequency of internet use (Figure 3). A Jonckheere-Terpstra test showed a statistically significant trend between lower eHEALS scores and decreasing internet use frequency (T_JT_=4861.5, *z*=−4.958; *P*<.001).

### Preferences in Communication

Almost all patients (198/200, 99%) wished for the opportunity to raise queries about complaints and treatment between routine medical visits. Preferred methods of contacting the treating gastroenterologist were telephone (81/200, 40.5%), email (61/200, 30.5%), and chat (56/200, 28%; Multimedia Appendix 5). Reminders for regular medication intake were requested frequently (172/200, 86%), with the favored mode being via an app (117/200, 58.5%). The vast majority of patients (196/200, 98%) wished to receive medical information, preferably through an app (92/200, 46%), followed by paper (62/200, 31%) and websites (42/200, 21%). If medical information was provided digitally, patients ranked simple text and images (172/200, 86%) over features based on exchanging information with other patients (20/200, 10%) and game-based learning (8/200, 4%). Acceptance of using a patient diary was high (176/200, 88%), with documentation preference being greatest for an app, followed by paper, and lowest for a website (Multimedia Appendix 5).

## Discussion

### Principal Findings and Comparison With Prior Work

To the best of our knowledge, this study is the first to provide an in-depth perspective on German patients’ views of mHealth solutions in gastroenterology, specifically in the context of IBD. Our findings emphasize that patients with IBD are interested in mHealth technologies and are willing and motivated to use them. For this intention to be realized, two factors are crucial: the availability of the necessary technical requirements, which was found to be remarkably high in our sample, with almost all patients (197/200, 98.5%) regularly using a smartphone and more than half (108/200, 54%) regularly using a tablet; and the possession of digital skills. On the basis of participants’ internet use behavior and eHealth literacy levels, it can be assumed that patients with IBD possess these skills. Nearly all patients (193/200, 96.5%) reported using the internet for health matters, underscoring its role as a critical source of health information. Patients in our study demonstrated the ability to find, evaluate, and use such information, as reflected by a mean eHEALS score of 28.9 (SD 5.4; range 8-40). This score indicates high eHealth literacy, considering that a previous study defined a cutoff for high eHealth literacy at ≥26 [29]. Numerous studies in various fields [21,29-33] have examined possible correlations between the dimensions of eHealth literacy and patient characteristics and behaviors. In our study of patients with IBD, younger age was associated with higher eHealth literacy, whereas no effect of sex was observed. Consistent with prior studies [21,29,30,33], lower eHealth literacy was associated with declining internet use frequency. Interestingly, belief in the usefulness of medical apps did not seem to depend on eHealth literacy levels but was overall high, highlighting patients’ widespread confidence in the potential of mHealth.

To fully realize this potential, most patients were willing to participate actively by inputting personal health data into IBD-specific apps, sharing them with their treating physicians, and providing them for research purposes. Such active participation ensures electronic data collection of a high standard, both in terms of quality and quantity, which can be of considerable benefit to patients and physicians alike. Prior studies [34-36] have also highlighted the usefulness of patient-generated health data. In addition, patients with IBD expressed clear preferences for digital interaction with their treating physicians, including consultations via video calls, filling out questionnaires online before appointments, and receiving electronic medical reports. These preferences could contribute to improved care by optimizing treatment processes. Despite the overall high enthusiasm for mHealth among patients with IBD, some preferred personal contact with their physician, as reflected by the highest preference for telephone calls for communication rather than eHealth services. Therefore, offering flexible communication options tailored to patient preferences is important. Furthermore, some of the patients (29/200, 14.5%) expressed reluctance to share data with their treating physicians due to concerns about security and transparency. These concerns must be taken seriously. The responsibility to guarantee data security lies with the various stakeholders involved and is critical not only to avoid undermining patients’ confidence in mHealth solutions but also to strengthen it. Further investigation into which specific data security features would make patients feel safe and strengthen their confidence is crucial. In addition, clear communication about data protection measures and transparent handling of data are essential to foster patient trust and encourage wider mHealth adoption.

mHealth apps have the potential to serve as powerful tools and are therefore of particular interest for research. As noted, patients’ belief in the usefulness of medical apps was remarkably high, and they were willing to actively use IBD-specific apps by inputting data for up to 5 minutes on a weekly basis. Desired features for an IBD-specific app differed, but preferences were primarily for those that deliver information, such as a newsletter about new scientific work and clinical studies, as well as electronic prescriptions. By contrast, interest in direct peer exchanges and integrated wearables was low, which is partially consistent with prior findings among patients with gastroenterological diseases [37]. Mobile apps can improve treatment adherence [38,39], and, in our study, patients with IBD expressed interest in features such as medication reminders and an app-based patient diary. Usability and security were also identified as key features of an IBD-specific app. Patient perspectives therefore provide valuable guidance for app design. Particular attention should be given to ensuring an intuitive user experience through simple, user-friendly design. App features should be customizable because preferences differ; however, features such as comprehensible information (eg, through simple text and images), electronic prescriptions, medication reminders, and patient diaries seem to be broadly desirable. In addition, the app should minimize demands on data input and guarantee data security so that patients feel safe using it. In line with previous recommendations [20,40,41], involving patients in the app development process is important, as it can enhance acceptance and use [41].

Despite the enormous interest and readiness of patients with IBD, current use of mHealth solutions remains relatively low. Only 12.5% (25/200) of the patients in our study reported using medical apps, of which just 2 were IBD specific (used by 3/25, 12%). Limited availability of IBD-specific apps, as noted in a previous study by our group [20], and the generally low knowledge of IBD-specific digital services likely represent two important factors contributing to the gap between patient interest and actual adoption. Further factors, such as usability concerns and skepticism regarding effectiveness, as reported in studies from other fields [42,43], require investigation in future research. Overall, the high discrepancy points to the need to develop effective mHealth solutions, supported by guidance and recommendations from gastroenterological specialists and societies regarding suitable and reliable sources and services and supported by qualitative patient education to build confidence in using these services, consistent with prior suggestions [42,44]. However, several challenges must be addressed; for example, strict regulations in Germany under the Digital Health Care Act (“Digitale-Versorgungs-Gesetz”), which classify prescribable health apps as medical devices and impose stringent data security demands [45], pose obstacles to the development and implementation of mHealth services. Nevertheless, translating these needs into practice to align with the interest and readiness of patients with IBD is going to be a crucial task and will require collaboration among all stakeholders to enable the implementation of reliable mHealth programs and apps in routine IBD care, thereby fully realizing the potential of mHealth.

### Limitations

The main limitations of this study include its cross-sectional design, which does not allow conclusions regarding the evaluation of patients’ digital engagement behaviors over time; reliance on self-reported data, which may be influenced by social desirability bias, potentially leading patients to overestimate their digital competencies or willingness to use mHealth tools; and a relatively small, circumscribed sample (outpatients from a single tertiary care center). Moreover, the relatively young mean age (39.1, SD 14.0 y) of the sample may influence findings, as older patients could exhibit different digital health behaviors and face distinct barriers to mHealth adoption. In addition, the number and characteristics of patients who declined participation were not recorded, limiting the assessment of potential selection bias. Therefore, the generalizability of the results may be compromised, as factors such as actual mHealth use could vary from what participants reported.

### Conclusions

To the best of our knowledge, this study is the first to comprehensively explore perspectives on mHealth among German patients with IBD. The insights gained have the potential to significantly influence and shape the development and integration of IBD apps. A vast majority of participants not only believed in the possible health benefits of using medical apps but were also motivated and willing to use mHealth technologies and even participate actively by inputting personal health data into apps and sharing them with physicians and researchers. Furthermore, fundamental requirements for successful mHealth use were largely met, as almost all patients regularly used smartphones and demonstrated considerable digital skills, as indicated by a high level of eHealth literacy. Despite these favorable conditions, current use of mHealth among patients with IBD remains limited, likely due to limited availability and awareness of existing apps, usability concerns, lack of perceived necessity, and skepticism about app effectiveness. To close this gap, it is crucial to develop effective mHealth solutions, supported by guidance and recommendations from gastroenterological specialists and societies regarding suitable and reliable sources and services, along with qualitative education on mHealth use. The unmet needs and patient priorities highlighted in this study provide direction for expediting the incorporation of mHealth into standard IBD care.

## Appendix

Multimedia Appendix 1 Original German questionnaire.

Multimedia Appendix 2 Patients’ viewpoints on medical apps.

Multimedia Appendix 3 Information on internet use, perceived benefit, knowledge of inflammatory bowel disease–specific digital offers, and eHealth literacy.

Multimedia Appendix 4 eHealth literacy measured with the eHealth Literacy Scale.

Multimedia Appendix 5 Preferences in communication.

## Abbreviations

eHEALS: eHealth Literacy Scale
IBD: inflammatory bowel disease
mHealth: mobile health
