# Supplementary material for: mHealth Use, Preferences, Barriers, and eHealth Literacy Among Patients With Inflammatory Bowel Disease: Survey Study
Source: JMIR Hum Factors. 2025 Nov 13;12:e64471. doi: 10.2196/64471 (PMC12661228; doi:10.2196/64471)
Supplement: Multimedia Appendix 1 [file humanfactors_v12i1e64471_app1.pdf]

**mHealth in Gastroenterology**

Universitätsklinikum Erlangen

Pat. Nr.: ____________ Datum: __ __ - __ __ - __ __
Tag Monat Jahr

**Liebe Patientinnen und Patienten, vielen Dank für Ihre Unterstützung!!**
**Bitte schreiben Sie leserlich in großen DRUCKBUCHSTABEN und füllen den Fragebogen komplett aus. Bitte überspringen Sie keine Fragen! Bei Fragen können Sie sich jederzeit an einen Mitarbeiter wenden!**

## Demographische Daten

1. **Alter:** _______ **Geschlecht:** ☐ männlich ☐ weiblich

2. **Chronisch-Entzündliche Darmerkrankung:**

   ☐ Morbus Crohn ☐ Colitis ulcerosa

3. **Wie aktiv ist die Krankheit im Moment (Patient Global)?**

| Eher inaktiv/0 | 1 | 2 | 3 | 4 | 5 | 6 | 7 | 8 | 9 | sehr aktiv/10 |
|---|---|---|---|---|---|---|---|---|---|---|
| ☐ | ☐ | ☐ | ☐ | ☐ | ☐ | ☐ | ☐ | ☐ | ☐ | ☐ |

4. **Jahr der Erstdiagnose:** ____________

5. **Was ist Ihr höchster Bildungsabschluss?**

   ☐ Lehre/ Berufsausbildung
   ☐ Fachhochschulabschluss
   ☐ Universitätsabschluss
   ☐ Ohne beruflichen Bildungsabschluss
   ☐ Bildungsabschluss noch nicht beendet

6. **Bitte geben Sie die Größe Ihres Wohnorts an (gemessen an der Einwohnerzahl):**

   ☐ Dorf (< 5.000)
   ☐ kleine Stadt (10.000-50.000)
   ☐ mittelgroße Stadt (50.000-100.000)
   ☐ große Stadt (> 100.000)

## E-Health Nutzung

7. **Benutzen Sie regelmäßig elektronische Geräte?**
   ☐ Smartphone (iPhone etc.)
   ☐ Tablet (iPad etc.)
   ☐ elek. Tracking Geräte (z.B. Fitnessarmbänder)
   ☐ weitere: ____________________________

8. **Nutzen Sie soziale Netzwerke (Facebook, Instagram, Twitter, etc.)?**
   ☐ Ja
   ☐ Nein

**9. Haben Sie schon mal Informationen über Gesundheitsthemen im Internet gesucht? (Mehrfachauswahl)**

☐ Ja →
- ☐ Diagnosen
- ☐ Symptome
- ☐ Medikamente
- ☐ Behandlungsmöglichkeiten
- ☐ Ärzte
- ☐ Patientenforen
- ☐ Krankheitsspezifische Websites
- ☐ Sonstiges: ______________________________

☐ Nein →
- ☐ Ich weiß nicht wie man diese Informationen im Internet sucht
- ☐ Ich habe keinen PC / Smartphone / Internetzugang
- ☐ Die Informationen von meinem Arzt waren ausreichend
- ☐ Ich denke nicht, dass mir das helfen würde.
- ☐ Sonstiges: ______________________________

**10. Wenn ja wie häufig nutzen Sie das Internet für med. Informationen?**

☐ täglich ☐ wöchentlich ☐ monatlich ☐ seltener ☐ gar nicht

**11. Haben Sie mit einem behandelnden Arzt schon mal per E-Mail kommuniziert?**

☐ Ja ☐ Nein

**12. Haben Sie schon mal ein Patientenforum genutzt und... (Mehrfachauswahl)?**

☐ einen aktiven Beitrag verfasst ☐ sich mit anderen Patienten ausgetauscht

☐ Beiträge gelesen ☐ weder noch

**13. Haben Sie schon mal an einem medizinischen Online Programm teilgenommen (Rauchentwöhnung, Stressreduktion, Schmerzreduktion)?**

☐ Ja ☐ Nein

**14. Kennen Sie die Website des Kompetenznetz Darmerkrankungen in Deutschland mit den Medikamenteninformationen für Patienten und Ärzte?**

☐ Ja ☐ Nein

**15. Kennen Sie hilfreiche CED-spezifische digitale Angebote (Websites/Apps)?**

☐ Ja, z.B. ____________________________________________

____________________________________________

☐ Nein

**16. Wie oft haben Sie folgende Möglichkeiten innerhalb der letzten 3 Monate genutzt um Gesundheitsinformationen zu erhalten?**

Bitte markieren Sie bei jeder Möglichkeit ihre Nutzung.

| | täglich | wöchentlich | mona tlich | seltener | gar nicht |
|---|---|---|---|---|---|
| Internet | ☐ | ☐ | ☐ | ☐ | ☐ |
| Medizinische Apps | ☐ | ☐ | ☐ | ☐ | ☐ |
| Printmedien (Broschüren, Bücher, etc.) | ☐ | ☐ | ☐ | ☐ | ☐ |
| medizinisches Fachpersonal | ☐ | ☐ | ☐ | ☐ | ☐ |
| Freunde / Bekannte / Familie | ☐ | ☐ | ☐ | ☐ | ☐ |

## App-Nutzung / Präferenzen

**17. Welche medizinischen Apps nutzen Sie?**

☐ Keine ☐ ____________________________________________

**18. Wenn Sie med. Apps nutzen, wie häufig nutzen Sie diese medizinischen Apps?**

☐ täglich ☐ wöchentlich ☐ monatlich ☐ seltener ☐ gar nicht

**19. Kennen Sie die App „Arzneimittel aktuell“?**

☐ Ja ☐ Nein

**20. Wie wichtig sind Ihnen bei einer medizinischen App folgende Punkte:**

Bitte markieren Sie bei jeder Aussage die Rubrik, die Ihre Meinung am besten beschreibt.

| | **unwichtig/0** | **1** | **2** | **3** | **4** | **5** | **6** | **7** | **8** | **9** | **sehr wichtig/10** |
|---|---|---|---|---|---|---|---|---|---|---|---|
| **Datensicherheit** | ☐ | ☐ | ☐ | ☐ | ☐ | ☐ | ☐ | ☐ | ☐ | ☐ | ☐ |
| **Design** | ☐ | ☐ | ☐ | ☐ | ☐ | ☐ | ☐ | ☐ | ☐ | ☐ | ☐ |
| **Benutzerfreundlichkeit** | ☐ | ☐ | ☐ | ☐ | ☐ | ☐ | ☐ | ☐ | ☐ | ☐ | ☐ |
| **Interaktionsmöglichkeit/ Unterhaltungswert** | ☐ | ☐ | ☐ | ☐ | ☐ | ☐ | ☐ | ☐ | ☐ | ☐ | ☐ |

**21. Ich glaube, dass der Einsatz von med. Apps hilfreich für mich sein kann**

☐ Ja ☐ Nein

**22. Ich würde es gut finden, mit meinem Arzt auch per Online Videosprechstunde kommunizieren zu können**

☐ Ja ☐ Nein

## eHealth Literarcy

**23. Wie nützlich/hilfreich finden Sie das Internet, um Entscheidungen über Ihre Gesundheit zu treffen?**

☐ überhaupt nicht nützlich/ hilfreich ☐ nicht nützlich/ hilfreich ☐ unsicher ☐ nützlich/ hilfreich ☐ sehr nützlich/ hilfreich

**24. eHEALS-Fragebogen:**

Bitte markieren Sie bei jeder Aussage die Rubrik, die Ihre Meinung am besten beschreibt.

| | trifft gar nicht zu | trifft nicht zu | neutral | trifft zu | trifft voll zu |
|---|---|---|---|---|---|
| Ich weiß, wie ich im Internet nützliche Gesundheitsinformationen finde. | ☐ | ☐ | ☐ | ☐ | ☐ |
| Ich weiß, wie ich das Internet nutzen kann, um Antworten auf meine Fragen rund um das Thema Gesundheit zu bekommen. | ☐ | ☐ | ☐ | ☐ | ☐ |
| Ich weiß, welche Quellen für Gesundheitsinformationen im Internet verfügbar sind. | ☐ | ☐ | ☐ | ☐ | ☐ |
| Ich weiß, wo im Internet ich nützliche Gesundheitsinformationen finden kann. | ☐ | ☐ | ☐ | ☐ | ☐ |
| Ich weiß, wie ich Informationen aus dem Internet so nutzen kann, dass sie mir weiterhelfen. | ☐ | ☐ | ☐ | ☐ | ☐ |
| Ich bin in der Lage, Informationen, die ich im Internet finde, kritisch zu bewerten. | ☐ | ☐ | ☐ | ☐ | ☐ |
| Ich kann im Internet zuverlässige von fragwürdigen Informationen unterscheiden. | ☐ | ☐ | ☐ | ☐ | ☐ |
| Wenn ich gesundheitsbezogene Entscheidungen auf Basis von Informationen aus dem Internet treffe, fühle ich mich dabei sicher. | ☐ | ☐ | ☐ | ☐ | ☐ |

## Kommunikationspräferenzen

**Welche Angebote würden Sie gerne nutzen, um sich über Ihre Erkrankung zu informieren und Ihren Therapieplan (z.B. Tabletteneinnahme) gut einhalten zu können?**

**25. Erinnerungshilfen**

Bitte ordnen Sie die angegebenen Möglichkeiten nach dem für Sie zutreffenden Stellenwert von 1 (höchster Stellenwert) bis 6 (niedrigster Stellenwert) gemäß Ihrer eigenen Meinung!

An die regelmäßige Tabletteneinnahme möchte ich erinnert werden per (Einordnung 1-6)

- SMS _______
- Anruf _______
- E-Mail _______
- Push-Nachrichten in einer App _______
- gar nicht _______
- Postkarte _______

**26. Informationen zur Erkrankung und zur Behandlung**

Bitte ordnen Sie die angegebenen Möglichkeiten nach dem für Sie zutreffenden Stellenwert von 1 (höchster Stellenwert) bis 4 (niedrigster Stellenwert) gemäß Ihrer eigenen Meinung!

Ich bevorzuge medizinische Informationen (Einordnung 1-4)

- auf Papier gedruckt (z.B. Broschüren) _______
- keine Zusatzinformationen gewünscht _______
- in einer App _______
- auf einer Website _______

**27. Informationsvermittlung zur Erkrankung und zur Behandlung bei digitalen Angeboten**

Bitte ordnen Sie die angegebenen Möglichkeiten nach dem für Sie zutreffenden Stellenwert von 1 (höchster Stellenwert) bis 3 (niedrigster Stellenwert) gemäß Ihrer eigenen Meinung!

Bei digitalen Angeboten (Website/App) wären mir wichtig (Einordnung 1-3)

- Info Text und Bilder _______
- spielerisches Lernen _______
- Austausch mit anderen _______

**28. Dokumentation von Beschwerden und der Tabletteneinnahme**

Bitte ordnen Sie die angegebenen Möglichkeiten nach dem für Sie zutreffenden Stellenwert von 1 (höchster Stellenwert) bis 4 (niedrigster Stellenwert) gemäß Ihrer eigenen Meinung!

Angaben zu meinem Befinden und zur Tabletteneinnahme würde ich in einem vorbereiten Formular („Patiententagebuch") dokumentieren (Einordnung 1-4)

- auf Papier gedruckt _______
- in einer App _______
- auf einer passwortgeschützten Website _______
- gar nicht _______

**29. Bei digitalen Angeboten (Website/App) wäre mir wichtig zu sehen, wie gut ich im Vergleich zu anderen Patienten den Therapieplan einhalte**

☐ wäre interessant zu wissen ☐ interessiert mich nicht

**30. Rückfrage-Möglichkeiten**

Bitte ordnen Sie die angegebenen Möglichkeiten nach dem für Sie zutreffenden Stellenwert von 1 (höchster Stellenwert) bis 4 (niedrigster Stellenwert) gemäß Ihrer eigenen Meinung!

Mit Fragen zu Beschwerden und zur Behandlung würde ich mich zwischen den Untersuchungsterminen gerne an die Ambulanz/ einen Arzt wenden mittels (Einordnung 1-4)

- Telefon _______
- E-Mail _______
- Website / Chat _______
- Keine Rückfragemöglichkeiten gewünscht _______

**31. Datenübermittlung an Arzt per App**

Würden Sie eine mobile App befürworten und Ihrem behandelnden Arzt Daten (Blutbild, Röntgenbilder, Arztbriefe etc.) zu Ihrer Erkrankung per App auf sicherem Weg zukommen lassen und Daten schnell zu erhalten?

☐ Ja

☐ Nein → (Mehrfachauswahl möglich)

☐ Ich habe keine passenden Endgeräte
☐ Ich kenne mich nicht mit der Technik aus
☐ Ich weiß nicht, was mit den Daten passiert
☐ Ich weiß nicht, wo die Daten gespeichert werden
☐ Ich befürchte keine gesicherte Datenübertragung
☐ Datenschutzgründe
☐ Ich möchte ausschließlich persönlichen Kontakt zum Arzt
☐ Ich halte es nicht für sinnvoll
☐ Andere Gründe: ________________________________________

**32. Wären Sie bereit eine aktive Dateneingabe (z.B. zu Lebensqualität, Gelenkstatus, etc.) über die App zu machen?**

☐ Ja → **Wie oft wären Sie zur aktiven Dateneingabe bereit?**

☐ täglich ☐ wöchentlich
☐ monatlich ☐ alle 3 Monate
☐ alle 6 Monate ☐ jährlich

→ **Wie viel Zeit würden Sie für die Dateneingaben aufwenden?**

☐ bis zu 5 Minuten ☐ 5-15 Minuten
☐ 15-30 Minuten ☐ länger

☐ Nein

**33. Würden Sie sich eine direkte Kontaktaufnahme bei Unregelmäßigkeiten Ihrer Eingaben (z.B. bei auffälligen Laborwerten, Ultraschall/Endoskopie Befund, starken Nebenwirkungen, etc.) wünschen?**

☐ Ja ☐ Nein

**34. Fänden Sie es gut, wenn Ihr betreuender Arzt bereits vor der Nachsorgeuntersuchung die eingegebenen Daten (Nebenwirkungen, Lebensqualität, Laborwerte) vorliegen hätte?**

☐ Ja ☐ Nein, weil ________________________________

**35. Würden Sie einer Verwendung Ihrer anonymisierten[1] oder pseudonymisierten[1] Daten zu Forschungszwecken (Verbesserung von Therapiemöglichkeiten, etc.) zustimmen?**

☐ Ja ☐ Nein,
weil____________________________________________________________________

**36. Ich würde die Patientenfragebögen auch gerne schon vor dem Termin per Internet/ PC/ Smartphone etc. ausfüllen können**

☐ Ja ☐ Nein

**37. Kennen Sie die offizielle App des Kompetenznetz Darmerkrankungen, MyTARGET©?**

☐ Ja ☐ Nein

**38. Ich wäre bereit auf einen Arztbrief in Papierform zu verzichten und „nur“ eine elektronische Version (z.B. als pdf) zu erhalten**

☐ Ja ☐ Nein

**39. Haben Sie weitere Vorschläge oder Ideen:________________________________________**

[1]Anonymisierung / Pseudonymisierung: Die Anonymisierung ist das Verändern personenbezogener Daten derart, dass diese Daten nicht mehr einer Person zugeordnet werden können. Bei der Pseudonymisierung wird der Name durch ein Pseudonym (zumeist eine mehrstellige Buchstaben- oder Zahlenkombination) ersetzt, um die Identifizierung des Betroffenen auszuschließen. Die Pseudonymisierung ermöglicht – unter Zuhilfenahme eines „Schlüssels“ – die Zuordnung von Daten zu einer Person. Diese Zuordnung ist ohne diesen Schlüssel nicht oder nur schwer möglich ist, da Daten und Identifikationsmerkmale getrennt sind.

**40. Wie sehr wären Sie an folgenden Funktionen einer CED-App interessiert?**

Bitte markieren Sie bei jeder Aussage die Rubrik, die Ihre Meinung am besten beschreibt.

| | interes siert mich sehr | interes siert mich | neutral | interes siert mich nicht | interes siert mich gar nicht |
|---|---|---|---|---|---|
| Visualisierung der Eingaben (Laborwerte, Verlauf, Nebenwirkungen, Krankheitsschübe, etc.) | ☐ | ☐ | ☐ | ☐ | ☐ |
| Dokumentation via Fotos | ☐ | ☐ | ☐ | ☐ | ☐ |
| Dokumentation von Stuhlfrequenz und -konsistenz, Bauschmerz, Gelenkschmerzen, Augen- und Hautveränderungen | ☐ | ☐ | ☐ | ☐ | ☐ |
| Erinnerungsfunktion (Infusionstermin, Medikamentengabe, Impfungen, regulärer Termin, Ausfüllen von Fragebögen, etc.) | ☐ | ☐ | ☐ | ☐ | ☐ |
| Mitteilungsmöglichkeit ob vor Infusionstermin ein Infekt vorliegt | ☐ | ☐ | ☐ | ☐ | ☐ |
| Dokumentation von Medikamenten | ☐ | ☐ | ☐ | ☐ | ☐ |
| Medikamenteninformationen/-warnungen (Nebenwirkungen, Wechselwirkungen, etc.) | ☐ | ☐ | ☐ | ☐ | ☐ |
| Informationen zu Ihrer Erkrankung | ☐ | ☐ | ☐ | ☐ | ☐ |
| Informationen zu Ernährung, Sportübungen, etc. | ☐ | ☐ | ☐ | ☐ | ☐ |
| wissenschaftlich fundierte Online-Übungen zur Reduktion von Schmerzen und Bewältigung von Stress | ☐ | ☐ | ☐ | ☐ | ☐ |

| | | | | | |
|---|---|---|---|---|---|
| elektronisches Rezept und elektronische AU-Bescheinigung | ☐ | ☐ | ☐ | ☐ | ☐ |
| Abklärung von Symptomen und Diagnosevorschläge, mittels intelligentem Algorithmus | ☐ | ☐ | ☐ | ☐ | ☐ |
| Updates zu neuen wissenschaftlichen Erkenntnissen zu Ihrer Erkrankung und Medikamenten/-studien | ☐ | ☐ | ☐ | ☐ | ☐ |
| Möglichkeit zum Austausch mit anderen Patienten | ☐ | ☐ | ☐ | ☐ | ☐ |
| passendes Equipment, z.B. Armband zur Datenerfassung der täglichen Bewegung (Schrittzähler), etc. | ☐ | ☐ | ☐ | ☐ | ☐ |
| Dokumentation von Infekten | ☐ | ☐ | ☐ | ☐ | ☐ |

Vielen Dank für die Teilnahme!
