# Supplementary figures and images for: mHealth Use, Preferences, Barriers, and eHealth Literacy Among Patients With Inflammatory Bowel Disease: Survey Study
Source: JMIR Hum Factors. 2025 Nov 13;12:e64471. doi: 10.2196/64471 (PMC12661228; doi:10.2196/64471)

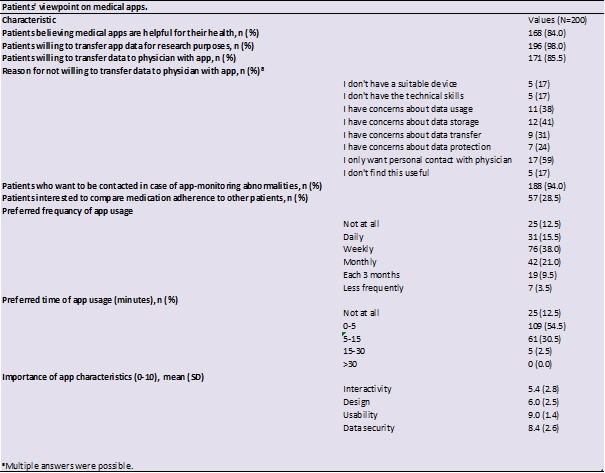

Supplement: Multimedia Appendix 2 [file humanfactors_v12i1e64471_app2.png]

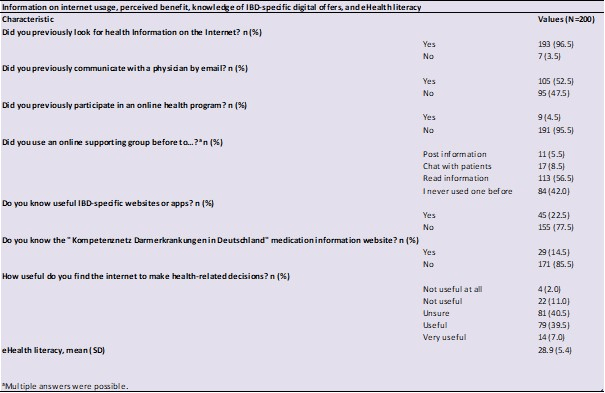

Supplement: Multimedia Appendix 3 [file humanfactors_v12i1e64471_app3.png]

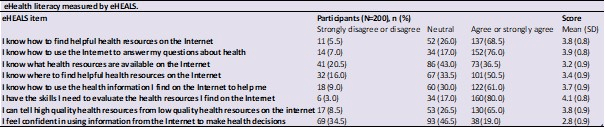

Supplement: Multimedia Appendix 4 [file humanfactors_v12i1e64471_app4.png]

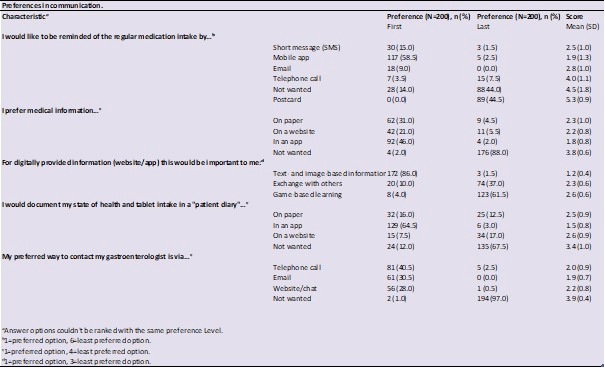

Supplement: Multimedia Appendix 5 [file humanfactors_v12i1e64471_app5.png]
